# Arthroscopic‐Assisted Suture Tape–Reinforced Triangular Fibrocartilage Complex Repair and Distal Radioulnar Ligament Augmentation Using a Modified Adams‐Berger Technique

**DOI:** 10.1002/atn2.70005

**Published:** 2026-04-28

**Authors:** Jin‐Ming Zhang, Hao‐Yang Tan, Xing‐Hao Deng, Shu‐Bo Li, Jian‐Zhong Xie, Yaxiang Liu, Zhong Chen

**Affiliations:** ^1^ Department of Sports Medicine Sun Yat‐sen Memorial Hospital Sun Yat‐sen University Guangzhou P. R. China; ^2^ Department of Sports Medicine Xingtan Hospital Affiliated to Shunde Hospital of Southern Medical University Foshan P. R. China; ^3^ Department of Sports Medicine Xiaolan People's Hospital of Zhongshan P. R. China; ^4^ Zhongshan School of Medicine Sun Yat‐sen University Guangzhou China

## Abstract

Distal radioulnar joint instability secondary to triangular fibrocartilage complex (TFCC) injury remains a major challenge in wrist surgery. Traditional tendon graft–based reconstruction techniques, such as the Adams‐Berger procedure, though effective, require extensive soft tissue dissection and tendon harvesting, resulting in significant surgical morbidity. Meanwhile, isolated arthroscopic TFCC repairs often fail to achieve durable stabilization in chronic or degenerative cases due to poor tissue quality and insufficient fixation strength. To overcome these limitations, we present a modified minimally invasive technique that combines arthroscopic TFCC repair using FiberTape with an anatomic augmentation of the distal radioulnar ligament based on the Adams‐Berger concept. A small volar incision is made over the distal radioulnar joint to create a bone tunnel at the radial insertion of the TFCC, through which FiberTape is introduced and routed along the volar and dorsal aspects of the joint capsule to the ulnar side. Under direct arthroscopic visualization, a suture lasso is used to shuttle the FiberTape through the TFCC and across its anatomic attachment at the ulnar fovea. Final tensioning and knot tying are performed at the ulnar tunnel outlet, forming a circumferential internal brace that anatomically replicates the deep distal radioulnar ligament configuration. This technique provides immediate biomechanical stability without the need for tendon harvesting, minimizes soft tissue disruption, and enables early postoperative mobilization. It offers a reproducible, low‐morbidity, and anatomically faithful method for distal radioulnar joint augmentation.

## Graphical abstract

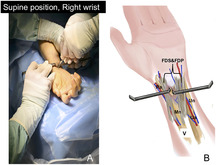

**VIDEO 1:** The procedure was performed with the patient in the supine position. The operative arm was placed horizontally on an arm board, abducted approximately 90 degrees from the torso at shoulder level, allowing optimal access to the wrist. Step 1: Volar‐Ulnar Approach and DRUJ Capsular Exposure: A 2 cm volar‐ulnar incision is made to expose the DRUJ capsule. Step 2: Radial Bone Tunnel Preparation and FiberTape Passage: A 2‐mm bone tunnel is drilled from the volar to dorsal aspect of the distal radius, along the anatomic trajectory of the distal radioulnar ligament. A spinal needle preloaded with PDS suture is inserted through the tunnel to shuttle the FiberTape across the sigmoid notch. Step 3: Subcapsular FiberTape Routing Along the DRUJ for Ulnar Exit: A 4‐cm incision is made along the distal ulna. The previously passed FiberTape is retrieved subcutaneously from both volar and dorsal sides of the distal radioulnar joint capsule, emerging through the ulnar incision in preparation for TFCC repair and ligament augmentation. Step 4: Arthroscopic‐Assisted Ulnar Tunnel Drilling. Step 5: Arthroscopic FiberTape Passage Through TFCC: Under arthroscopic visualization, a spinal needle is used to pass a PDS suture through the peripheral portion of the TFCC. The suture then guides the dorsal and volar limbs of the FiberTape through the TFCC and out via the ulnar styloid tunnel. Step 6: Final Tensioning and Multi‐Knot Volar Fixation: With the wrist in neutral or slight supination, the volar and dorsal limbs of the FiberTape are tensioned evenly, each encircling the ulna. The two limbs are then tied together on the volar side of the ulna with four secure knots. (DRUJ, distal radioulnar joint; PDS, polydioxanone suture; TFCC, triangular fibrocartilage complex.) Video content can be viewed at https://doi.org/10.1002/atn2.70005.

Distal radioulnar joint (DRUJ) instability is a functionally disabling condition frequently caused by injury to the triangular fibrocartilage complex (TFCC), particularly its deep radioulnar ligament components.^1^ Traditional anatomical reconstruction techniques, such as the Adams‐Berger procedure, have shown favorable clinical outcomes but typically require tendon graft harvesting and extensive open exposure.

The emergence of FiberTape‐based internal bracing has provided surgeons with a promising alternative, enabling ligament reinforcement without the morbidity associated with graft harvesting.^2^ Concurrently, advances in wrist arthroscopy now permit direct visualization of intra‐articular pathology and facilitate more accurate, minimally invasive soft tissue repair.

Classical DRUJ ligament reconstructions, including the Adams‐Berger technique, are commonly indicated for chronic instability in patients without osseous deformities or advanced arthritis. Despite their efficacy, these procedures require significant soft tissue dissection, including opening of both volar and dorsal capsules and transosseous drilling at the ulnar fovea. These steps pose risks of disrupting TFCC vascularity, damaging the native ligamentous envelope, and increasing postoperative stiffness, pain, and sensory disturbances.^3^ In addition, conventional tendon grafts do not reliably replicate the layered anatomical and biomechanical properties of the deep and superficial distal radioulnar ligaments (DRULs). Friction between the graft and bone tunnel may also result in degeneration or iatrogenic fracture over time.

Although arthroscopic foveal TFCC repairs have yielded satisfactory results in selected patients, outcomes are often suboptimal in chronic or grossly unstable cases where tissue quality is compromised by fibrosis or attrition.^4^ In such scenarios, isolated soft tissue repair may be insufficient to restore rotational stability.

To address these limitations, we describe a minimally invasive, anatomically guided modification of the Adams‐Berger technique, integrating FiberTape (Arthrex, Naples, FL) reinforcement with arthroscopic‐assisted TFCC repair. This technique preserves capsular integrity, eliminates the need for tendon harvest, provides reliable fixation, and supports early postoperative rehabilitation, offering a lower‐morbidity, anatomically precise alternative for patients with DRUJ instability.

## SURGICAL TECHNIQUE

Arthroscopic‐assisted suture tape–reinforced TFCC repair and DRUL augmentation is described in a step‐by‐step manner below and is shown in the Video 1. Pearls and pitfalls are presented in Table 1. Advantages and disadvantages are given in Table 2.

**TABLE 1:** Pearls and Pitfalls

Pearls	Pitfalls
Use arthroscopic visualization to accurately create 2.0‐mm bone tunnels in both the radius and ulna, minimizing the risk of tunnel over enlargement commonly seen in traditional tendon graft techniques	Overtightening the FiberTape may overconstrain the distal radioulnar joint and restrict forearm rotation
Employ a curved clamp to guide the FiberTape along the volar and dorsal capsules of the distal radioulnar joint, ensuring it exits precisely through the ulnar incision	Misplacement of the ulnar or radial tunnels may result in nonanatomic reconstruction and persistent instability
Maintain the wrist in a neutral or slightly supinated position during final tensioning to ensure balanced augmentation	Inadequate arthroscopic skill or portal placement may limit visualization and compromise repair accuracy
Route the FiberTape dorsal and volar to the triangular fibrocartilage complex to mimic native deep ligament orientation	Failure to protect neurovascular structures during volar and ulnar exposure may result in injury
Tie the knot on the volar side for easier access and lower risk of dorsal irritation	

**TABLE 2:** Advantages and Disadvantages

Advantages	Disadvantages
The technique enables anatomical restoration of the distal radioulnar ligaments without requiring tendon graft harvest	The procedure requires advanced arthroscopic skills and meticulous surgical technique, presenting a steep technical learning curve
A minimally invasive approach significantly reduces soft tissue trauma while preserving the structural integrity of the volar and dorsal capsules	Overtensioning of the FiberTape construct may result in overconstraint of the distal radioulnar joint, potentially impairing forearm rotational kinematics
FiberTape provides immediate biomechanical stabilization, allowing for early postoperative mobilization and rehabilitation	Long‐term clinical outcomes and durability data remain limited compared with traditional tendon graft–based reconstructions
The use of 2‐mm‐diameter bone tunnels minimizes cortical disruption and preserves osseous integrity compared with traditional tendon graft techniques that require larger tunnels. This reduces the risk of tunnel widening, stress fractures, and long‐term graft wear, while providing sufficient fixation strength for FiberTape‐based constructs	Postoperative complications may include local irritation or discomfort due to subcutaneous knot prominence or implant positioning
The biomechanical reinforcement system replicates the tension characteristics and spatial orientation of the native deep radioulnar ligament layers, enhancing rotational stability while minimizing graft‐related complications	

### Position and Setup

The procedure was performed with the patient in the supine position. The right upper limb was placed horizontally on an arm board, with the shoulder abducted to approximately 90° to allow optimal access to the wrist. Standard wrist arthroscopy portals were established, with the 3‐4 portal used as the viewing portal and the 6R portal used as the working portal.

**FIGURE 1:**
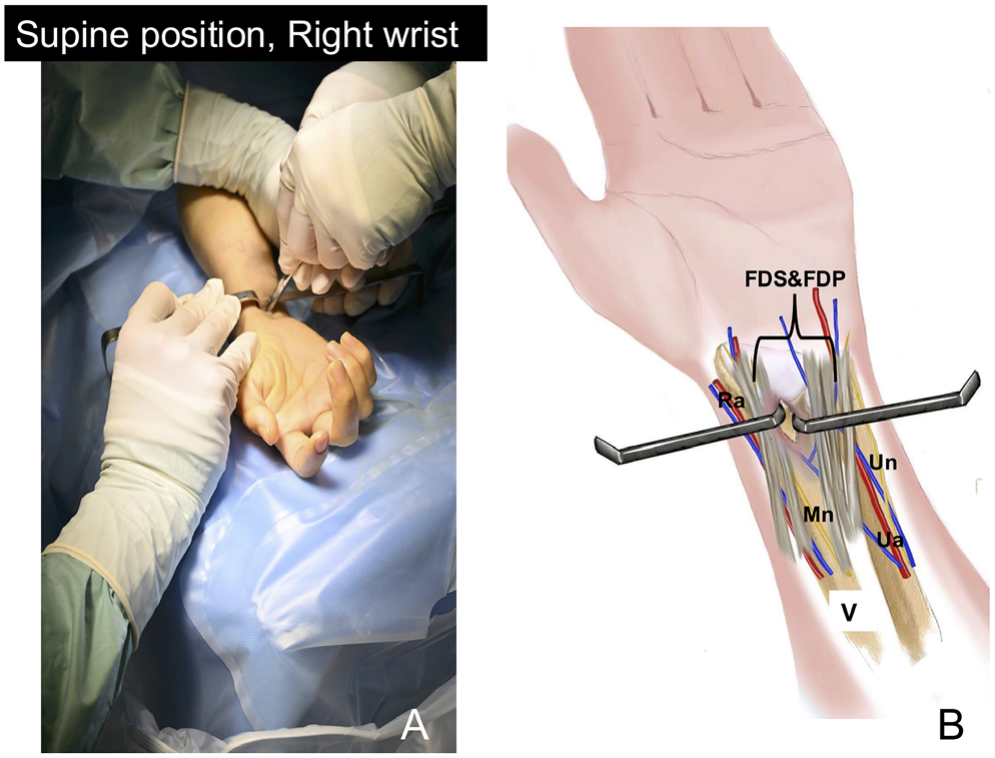
(A) A 2‐cm longitudinal incision is made along the volar‐ulnar aspect of the wrist to expose the volar capsule of the distal radioulnar joint (DRUJ). Critical neurovascular structures—including the ulnar nerve, median nerve, ulnar artery, and vein—as well as the flexor tendons are meticulously identified, protected, and retracted radially. (B) Schematic illustration showing the volar‐ulnar approach and the location of surrounding anatomical structures, highlighting the safe corridor for capsule exposure. (FDP, flexor digitorum profundus tendons; FDS, flexor digitorum superficialis tendons; Mn, median nerve; Ra, radial artery; Ua, ulnar artery; Un, ulnar nerve; V, volar.)

**FIGURE 2:**
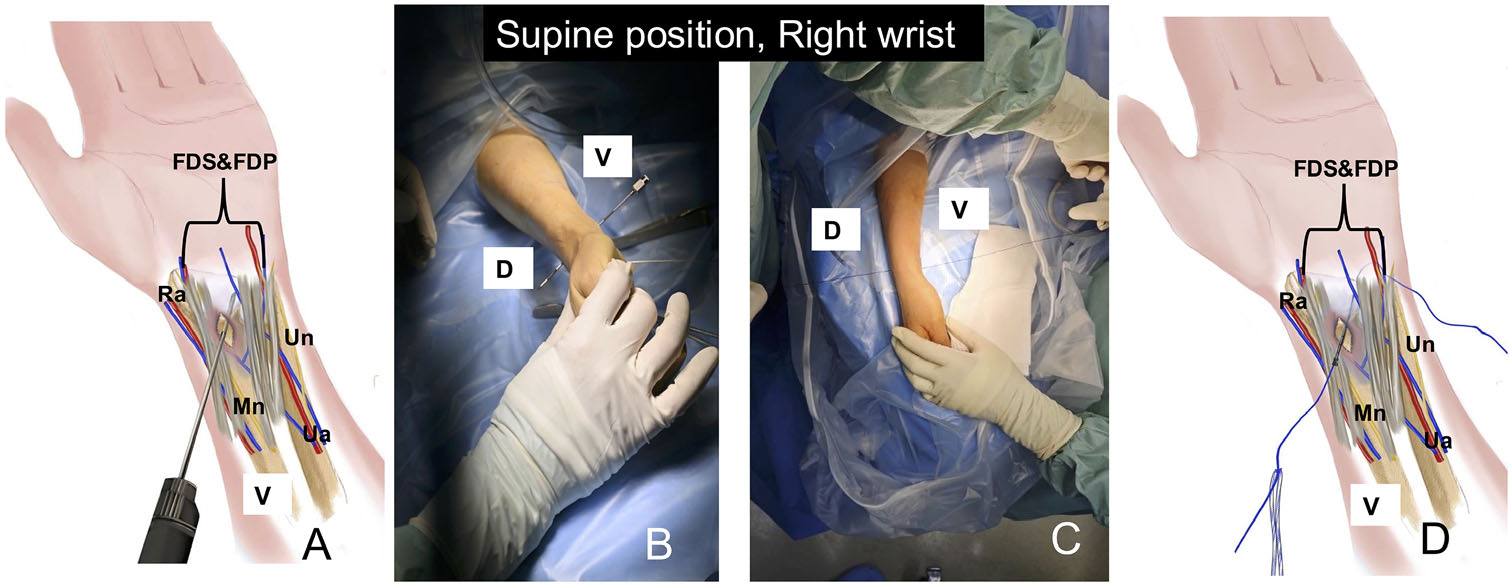
(A) Schematic illustration showing the creation of a 2.0‐mm bone tunnel from the volar to dorsal aspect of the distal radius, aligned with the anatomical trajectory near the sigmoid notch. (B) Insertion of the spinal needle into the radial tunnel, followed by its removal after placement of the PDS suture. (C) The PDS suture remains within the tunnel, allowing subsequent introduction of the FiberTape from the volar aspect. (D) Corresponding schematic showing volar‐to‐dorsal passage of the FiberTape through the bone tunnel using the PDS suture as a shuttle. (D, dorsal; FDP, flexor digitorum profundus tendons; FDS, flexor digitorum superficialis tendons; Mn, median nerve; Ra, radial artery; Ua, ulnar artery; Un, ulnar nerve; V, volar.)

**FIGURE 3:**
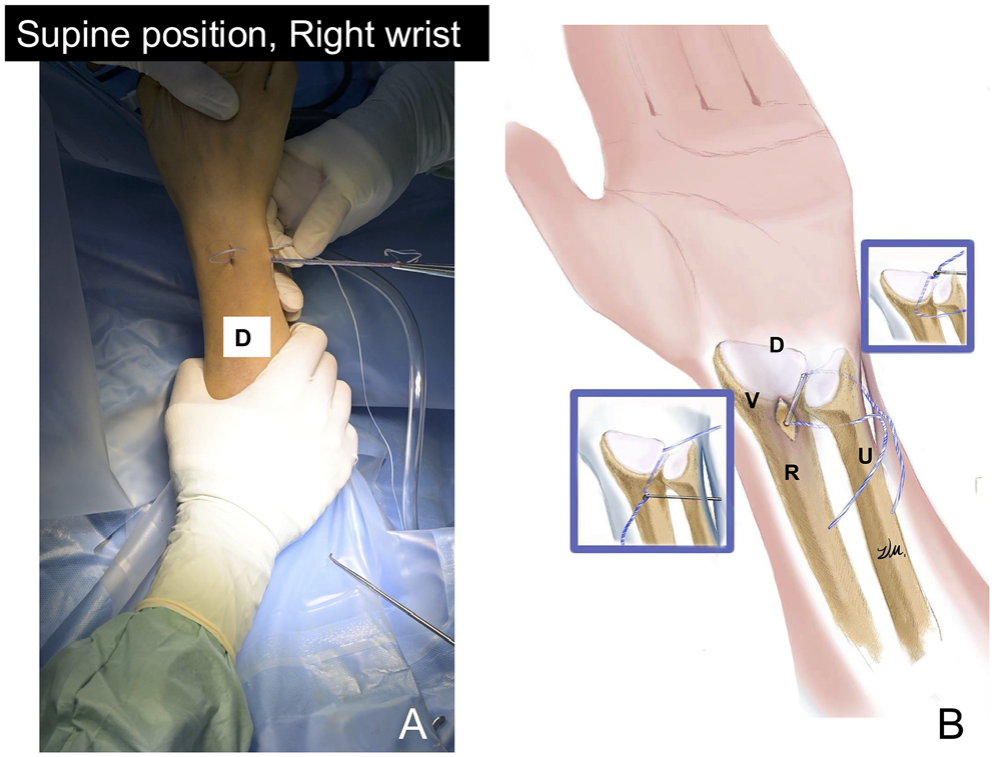
(A) The FiberTape is retrieved through a separate 4‐cm longitudinal ulnar incision, passed subcutaneously and closely abutting the volar and dorsal capsules of the distal radioulnar joint (DRUJ). This trajectory faithfully mimics the anatomic course of the deep distal radioulnar ligament while minimizing soft tissue disruption. (B) Schematic illustrating the FiberTape emerging from the ulnar incision along the volar and dorsal joint capsule surfaces, accurately positioned for subsequent trans‐TFCC ligament augmentation. (D, dorsal; R, radius; U, ulna; V, volar.)

**FIGURE 4:**
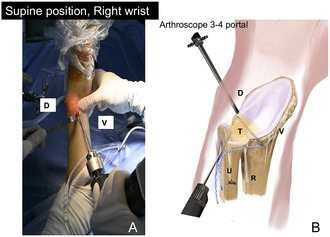
(A) Under wrist arthroscopic visualization via standard 3‐4 and 6R portals, a 2.0‐mm K‐wire is introduced approximately 5 mm proximal to the tip of the ulnar styloid to facilitate creation of a bone tunnel along the anatomical trajectory of the distal radioulnar ligament (DRUL). (B) Schematic illustration showing the K‐wire advanced at a 30° angle toward the foveal insertion of the DRUL, enabling precise tunnel placement while minimizing the risk of iatrogenic TFCC injury under arthroscopic guidance. (D, dorsal; R, radius; T, triangular fibrocartilage complex; U, ulna; V, volar.)

**FIGURE 5:**
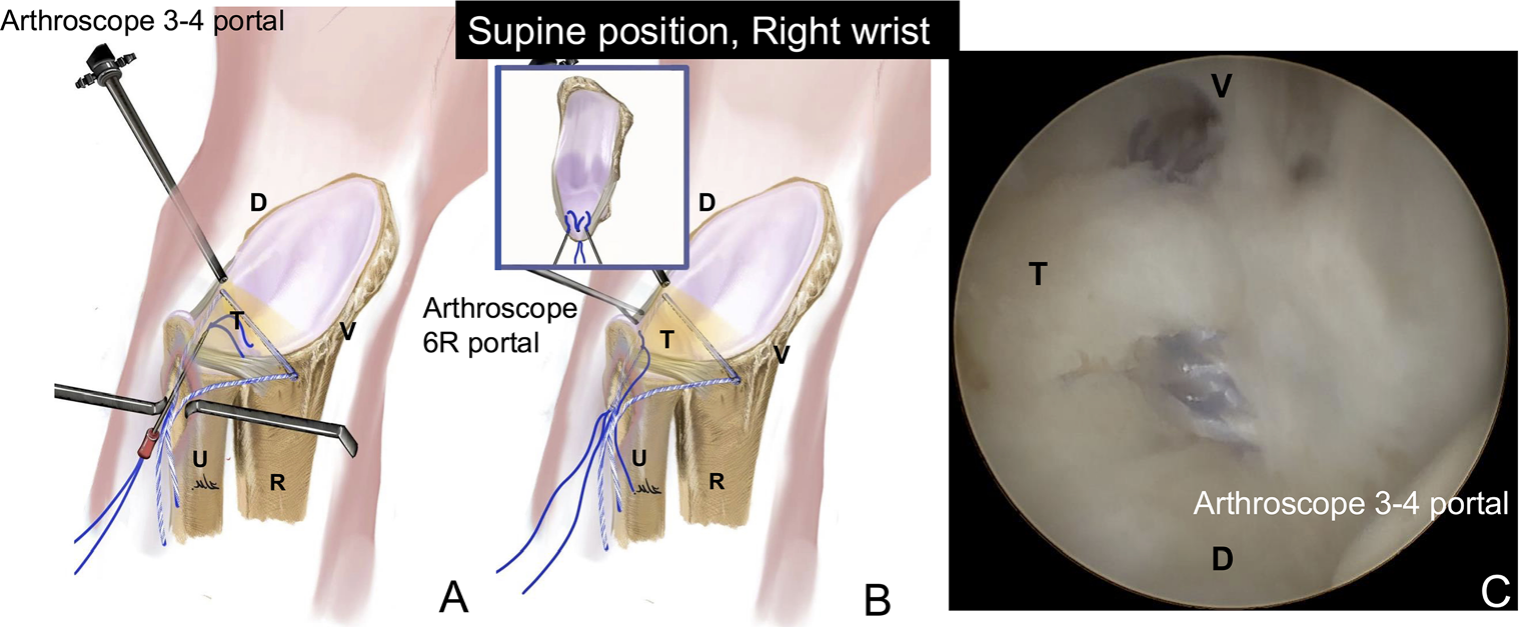
(A) Schematic illustration of a spinal needle passed through the peripheral margin of the triangular fibrocartilage complex (TFCC), with subsequent insertion of a PDS suture. (B) The FiberTape is shuttled through the triangular fibrocartilage complex (TFCC) using a PDS suture, following the anatomic trajectory of the distal radioulnar ligament (DRUL). The PDS suture is introduced and retrieved via the 6R portal, facilitating transosseous passage. Both volar and dorsal limbs of the FiberTape exit through the ulnar styloid bone tunnel. (C) Arthroscopic view from the 3‐4 portal of a right wrist in the supine position. The dorsal (D) and volar (V) limbs of the FiberTape are seen traversing the triangular fibrocartilage complex (TFCC) and exiting through the ulnar styloid bone tunnel. (D, dorsal; R, radius; T, triangular fibrocartilage complex; U, ulna; V, volar.)

**FIGURE 6:**
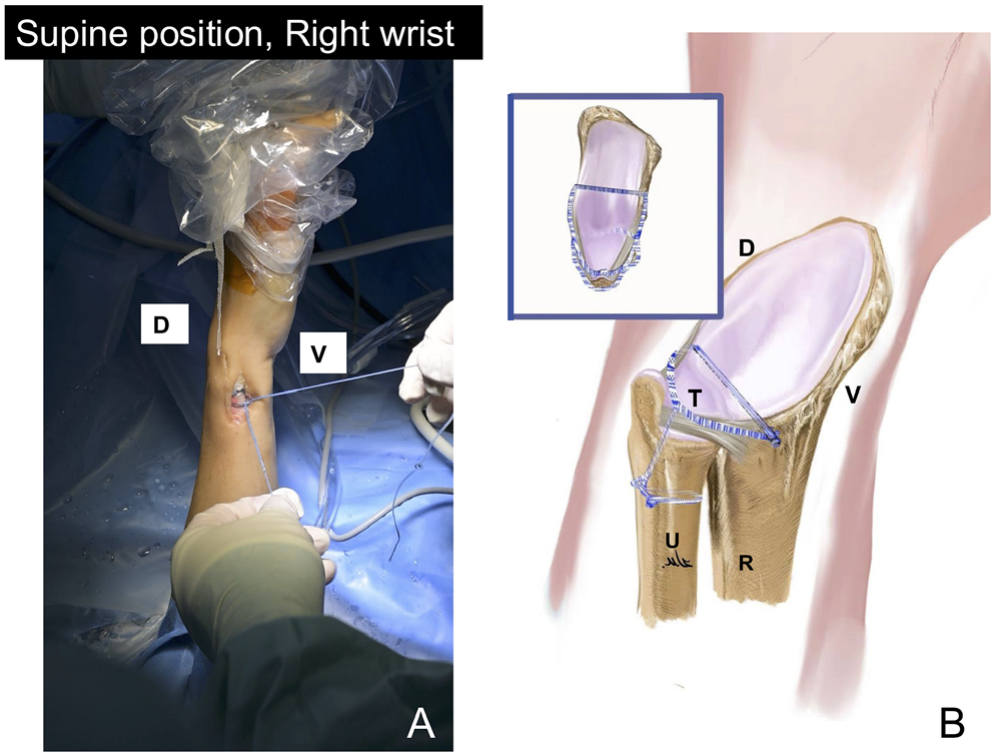
(A) With the wrist positioned in neutral or slight supination, the FiberTape is circumferentially tensioned around the ulna, and secure knot fixation is performed on the volar aspect. (B) Schematic illustration showing the final configuration and fixation technique. (D, dorsal; R, radius; T, triangular fibrocartilage complex; U, ulna; V, volar.)

1.
**Volar‐Ulnar Approach and DRUJ Capsular Exposure**
  A longitudinal incision approximately 2 cm in length is made along the volar‐ulnar aspect of the wrist. Sharp and blunt dissection is performed in layers to expose the volar capsule of the DRUJ. During the exposure, critical neurovascular structures—including the ulnar nerve, median nerve, ulnar artery, and vein—as well as the surrounding flexor tendons are carefully protected. These structures are gently retracted to both sides to ensure safe surgical access and avoid iatrogenic injury (Figure 1).
2.
**Radial Bone Tunnel Preparation and FiberTape Passage**
  A 2‐mm bone tunnel is drilled from the volar to the dorsal aspect of the distal radius, near the sigmoid notch, following the anatomic trajectory of the DRUL to replicate its native insertion. A spinal needle is introduced into the tunnel, preloaded with a Polydioxanone suture (PDS) suture, to facilitate the passage of FiberTape through the prepared radial tunnel (Figure 2).
3.
**Subcapsular FiberTape Routing Along the DRUJ for Ulnar Exit**
  A 4‐cm longitudinal incision is made along the distal subcutaneous border of the ulna. The previously passed FiberTape is routed subcutaneously across the volar and dorsal surfaces of the DRUJ capsule, emerging through the ulnar incision. This positions the FiberTape in preparation for subsequent TFCC repair and ligament augmentation (Figure 3).
4.
**Arthroscopic‐Assisted Ulnar Tunnel Drilling**
  Standard dorsal wrist arthroscopy portals (3‐4 and 6R) are established to access the joint. Under direct arthroscopic visualization, a 2.0‐mm Kirschner wire is introduced approximately 5 mm proximal to the tip of the ulnar styloid. The wire is advanced at an angle of approximately 30° relative to the long axis of the ulna to follow the anatomical trajectory toward the foveal attachment of the DRUL. Continuous arthroscopic monitoring throughout the procedure allows for precise tunnel placement while minimizing the risk of injury to the TFCC and adjacent crpal articular cartilage (Figure 4).
5.
**Arthroscopic FiberTape Passage Through TFCC**
  Under arthroscopy, a spinal needle is used to shuttle a PDS suture through the peripheral tissue of the TFCC. The PDS suture is then used to guide both the dorsal and volar limbs of the FiberTape through the TFCC and out through the external aperture of the ulnar styloid bone tunnel. The trajectory of the FiberTape is carefully designed to replicate the native anatomical orientation of the deep DRUL, thereby restoring joint stability (Figure 5).
6.
**Final Tensioning and Multiknot Volar Fixation**
  With the wrist held in a neutral position or slight supination, both limbs of the FiberTape are evenly tensioned to achieve appropriate compression across the reconstructed ligament pathway. The limbs are looped volarly around the ulna and sequentially secured using a series of 4 surgical knots to enhance fixation strength and minimize slippage. This technique achieves an anatomic tensioning profile while avoiding overconstraint of the DRUJ (Figure 6).

## DISCUSSION

DRUJ instability, particularly that arising from disruption of the deep fibers of the TFCC, presents a complex clinical challenge due to its implications on forearm rotation and load transmission across the wrist. The present technique integrates arthroscopic‐assisted TFCC repair with a modified Adams‐Berger–based ligament augmentation using FiberTape, representing a minimally invasive yet biomechanically robust solution for DRUJ stabilization.

Compared with traditional tendon graft–based reconstructions, which often require extensive capsular dissection and violate both dorsal and volar compartments, our technique preserves the capsuloligamentous envelope while achieving anatomical realignment of the DRULs.^5^ The use of dual 2.0‐mm bone tunnels at the anatomical insertion sites on the radius and ulna minimizes iatrogenic bone compromise relative to larger‐diameter tunnels required for autograft passage. Moreover, avoidance of tendon harvest reduces donor‐site morbidity and operative time, making the procedure both efficient and tissue‐sparing.

FiberTape offers distinct mechanical advantages. Its high tensile strength and minimal elongation under cyclic loading allow it to replicate the load‐bearing function of the native ligament.^6^ When routed circumferentially across the volar and dorsal joint capsule and passed arthroscopically through the TFCC at its ulnar foveal insertion, it simulates the trajectory of the deep DRUL. This construct not only provides immediate stabilization but also supports early initiation of postoperative motion protocols, which is critical to preventing adhesions and loss of forearm pronation‐supination.^7^

The technical reproducibility of this method is enhanced by arthroscopic visualization, which facilitates concurrent assessment of intra‐articular pathology and precise orientation of bone tunnels. Nonetheless, this approach requires proficiency in wrist arthroscopy and an intimate understanding of the 3‐dimensional DRUJ anatomy. Excessive FiberTape tensioning could lead to overconstraint and loss of physiological rotation, a complication mitigated in our method by neutral or slightly supinated positioning.^8^, ^9^

Limitations of this technique include its steep learning curve and the current lack of long‐term comparative data versus conventional tendon reconstructions. Although preliminary biomechanical studies suggest favorable load‐to‐failure and stiffness characteristics for FiberTape constructs, prospective clinical trials are warranted to evaluate durability, proprioceptive restoration, and complication rates such as knot irritation or tunnel osteolysis.^6^, ^10^ Furthermore, the implications of synthetic constructs on synovial fluid interaction and long‐term tissue remodeling require ongoing investigation.

This technique provides a minimally invasive and anatomically guided solution for restoring DRUJ stability in chronic TFCC injuries. By combining arthroscopic visualization with FiberTape reinforcement, it replicates native ligament structure while minimizing soft tissue disruption. The approach avoids tendon harvesting, reduces morbidity, and enables early rehabilitation, making it a safe, effective, and reproducible option for DRUJ augmentation.

## DISCLOSURES

The authors (J‐M.Z., H‐Y.T., X‐H.D., S‐B.L., J‐Z.X., Y.L., Z.C.) declare the following financial interests/personal relationships, which may be considered as potential competing interests: J‐M.Z. reports that financial support was provided by the National Natural Science Foundation of China‐Guangdong Joint Fund. H‐Y.T. reports that financial support was provided by the National Natural Science Foundation of China‐Guangdong Joint Fund. X‐H.D. reports that financial support was provided by the National Natural Science Foundation of China‐Guangdong Joint Fund. S‐B.L. reports that financial support was provided by the National Natural Science Foundation of China‐Guangdong Joint Fund. J‐Z.X. reports that financial support was provided by the National Natural Science Foundation of China‐Guangdong Joint Fund. Y.L. reports that financial support was provided by the National Natural Science Foundation of China‐Guangdong Joint Fund. Z.C. reports that financial support was provided by the National Natural Science Foundation of China‐Guangdong Joint Fund.

## FUNDING

This study was supported by the Natural Science Foundation of Guangdong Province (2022A1515011714).
